# Quantitative Trait Locus Mapping for Drought Tolerance in Soybean Recombinant Inbred Line Population

**DOI:** 10.3390/plants10091816

**Published:** 2021-08-31

**Authors:** Sanjeev Kumar Dhungana, Ji-Hee Park, Jae-Hyeon Oh, Beom-Kyu Kang, Jeong-Hyun Seo, Jung-Sook Sung, Hong-Sik Kim, Sang-Ouk Shin, In-Youl Baek, Chan-Sik Jung

**Affiliations:** 1 Department of Southern Area Crop Science, Upland Crop Breeding Research Division, National Institute of Crop Science, Rural Development Administration, Miryang 50424, Korea; sanjeev@korea.kr (S.K.D.); hellobk01@korea.kr (B.-K.K.); next0501@korea.kr (J.-H.S.); sjs31@korea.kr (J.-S.S.); shinso32@korea.kr (S.-O.S.); baekiy@korea.kr (I.-Y.B.); jung100@korea.kr (C.-S.J.); 2 Department of Agricultural Biotechnology, Gene Engineering Division, National Institute of Agricultural Sciences, Rural Development Administration, Jeonju 54874, Korea; jhoh8288@korea.kr; 3 Department of Central Area Crop Science, Crop Post-Harvest Technology Division, National Institute of Crop Science, Rural Development Administration, Suwon 16429, Korea; kimhongs@korea.kr

**Keywords:** candidate gene, quantitative trait locus, recombinant inbred line, soybean drought tolerance, weighted drought coefficient

## Abstract

Improving drought stress tolerance of soybean could be an effective way to minimize the yield reduction in the drought prevailing regions. Identification of drought tolerance-related quantitative trait loci (QTLs) is useful to facilitate the development of stress-tolerant varieties. This study aimed to identify the QTLs for drought tolerance in soybean using a recombinant inbred line (RIL) population developed from the cross between a drought-tolerant ‘PI416937’ and a susceptible ‘Cheonsang’ cultivar. Phenotyping was done with a weighted drought coefficient derived from the vegetative and reproductive traits. The genetic map was constructed using 2648 polymorphic SNP markers that distributed on 20 chromosomes with a mean genetic distance of 1.36 cM between markers. A total of 10 QTLs with 3.52–4.7 logarithm of odds value accounting for up to 12.9% phenotypic variance were identified on seven chromosomes. Five chromosomes—2, 7, 10, 14, and 20—contained one QTL each, and chromosomes 1 and 19 harbored two and three QTLs, respectively. The chromosomal locations of seven QTLs overlapped or located close to the related QTLs and/or potential candidate genes reported earlier. The QTLs and closely linked markers could be utilized in maker-assisted selection to accelerate the breeding for drought tolerance in soybean.

## 1. Introduction

Soybean (*Glycine max* [L.] Merr.) is one of the major commodity crops worldwide for food and feed sources (http://faostat.fao.org/). Increment in the production of major crops is crucial for global food security. However, the yield of many crops, including soybean, is challenged by global climate change [1]. Climate changes exacerbate the incidence of extreme weather patterns, such as erratic rainfall, elevated temperature, and the consequent drought stress, causing significant reductions in crop production [2]. Drought stress is a major abiotic stress that may cause more than 50% yield reduction in soybean [3]. Sensitivity of soybean plants to drought stress affects the global soybean yield because nearly 41% of the world’s land is dryland [4], and unpredictable climatic variability, including increased drought events, is experienced in many parts [5,6]. Although the negative influence of drought on soybean depends on the severity, duration, and timing of the stress about the growth stage, the most susceptible stage to drought stress is the reproductive stage [7,8]. Therefore, acquisition of genetic information on drought tolerance at the reproductive stages of soybean is of great importance.

Low soil water availability brings several physiological and biochemical changes in soybean plants that may induce a wide range of injury symptoms, such as reduced photosynthesis [9,10], increased oxidative stress [11], and alterations in metabolism [12]. These changes are reflected in various visible traits, including reduced plant height, the number of nodes, branches and pods, biomass, and leaf area in soybean [13,14,15]. As drought tolerance is a complex quantitative trait controlled by multiple genes [16], it can be expected that several traits and loci are associated with the ability to tolerate water-deficit stress in soybean. Therefore, the quantitative trait locus (QTL) studies for drought tolerance comprising traits like plant height, the number of nodes, branches and pods, biomass, and leaf area could be of high significance.

Identification of the genomic regions associated with drought tolerance can help accelerate soybean genetic research and varietal improvement. A few linkage mapping studies have been carried out to identify QTLs related to drought tolerance in soybean considering different traits. For instance, QTLs have been detected using seed yield and drought susceptibility [17], leaf wilting coefficient, excised leaf water loss, relative water content and seed yield [18], the conditioning of fibrous roots that is related to drought avoidance [19], water use efficiency and leaf ash [20,21], beta and carbon isotope discrimination [22], canopy wilting [23], and plant height and seed yield [24]. Recently, Wang et al. [25] used a genome-wide association study to identify QTL for drought tolerance considering the relative plant height and plant weight. 

One of the major limiting factors in the genetic study of drought tolerance was the availability of low-density markers, thereby reducing the efficiency and accuracy of QTL mapping. However, the rapid development of sequencing techniques has provided powerful tools like single nucleotide polymorphism (SNP) genotyping, enabling the development of the highest map resolution compared to other marker systems [26,27]. SNP markers have been used to discover QTL in many crops, including rice, maize, wheat, soybean, canola, barley, sugar beet, and cowpea [28]. Similarly, selection and measurements of relevant traits are equally important to precisely identify QTLs for stress tolerance. In this study, we considered a few vegetative as well as reproductive traits, such as plant height (PH), the number of nodes on the main stem (NN), branches (BN) and pods (PN), biomass (BM), and leaf area (LA) for phenotyping and SNP markers for genotyping the RIL population to identify QTL for drought tolerance. As these six traits are regarded as highly affected traits due to drought stress [13,14,15], this study provides valuable information on genetic understanding and breeding for drought tolerance in soybean.

## 2. Results

### 2.1. Soil Moisture Content

The soil moisture content of the control and treatment plots differed across three years according to the irrigation applied to the plots. On average, the control plots had 10–13% and the drought treatment plots had 3–10% soil moisture content. In 2017, the control plot showed an average of 11% and the treated plot showed an average of 7% soil moisture content. In 2018, the soil moisture content was 12.7 and 9.7% in the control and drought-treated plots, respectively. Similarly, the control plot showed 10% and the treated plot showed 3% moisture content in 2019.

### 2.2. Phenotypic Analysis of The Parents and 140 RILs

**Figure 1:**
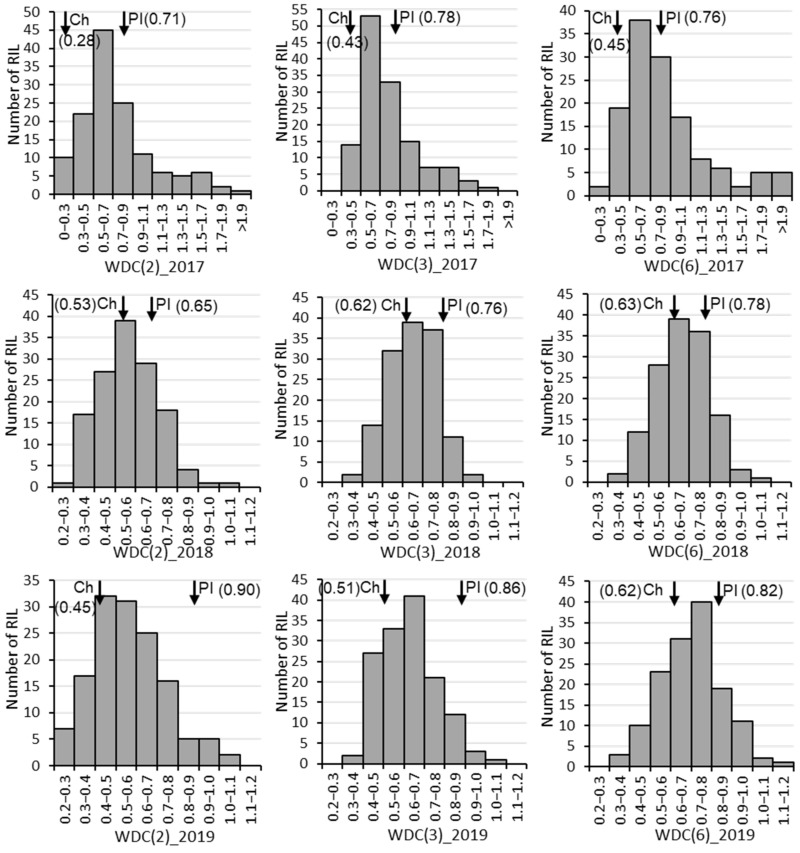
Frequencies in recombinant inbred line number for weighted drought coefficient (WDC) from 2017 to 2019. Ch and PI next to the inverted arrow (↓) with WDC value inside parentheses are abbreviated for the parents ‘Cheonsang’ and ‘PI416937’, respectively. The values in the parentheses after WDC indicate the number of traits considered to calculate WDC: 2 (biomass and leaf area), 3 (plant height, biomass, and leaf area), and 6 (plant height, node number, branch number, pod number, biomass, and leaf area).

The drought-tolerant parent ‘PI416937’ had consistently higher weighted drought coefficient (WDC) than the susceptible parent ‘Cheonsang’ for all three combinations of traits (Table 1). The mean WDC, calculated using two, three, and six traits, of ‘PI416937’ was 0.76, 0.80, and 0.79 and that of ‘Cheonsang’ was 0.42, 0.52, and 0.57, respectively. The highest WDC for ‘PI416937’ and ‘Cheonsang’ was found in 2019 and 2018, respectively. On the other hand, the highest WDC for the RILs was found in 2017. RIL distribution for WDC over three years showed normal distribution with transgressive segregation (Figure 1).

### 2.3. Linkage Mapping and QTL Analysis

The 19,259 polymorphic markers were binned (segregation distortion *p* < 0.001 and missing data with >15%) to eliminate the redundant markers. After binning, 2702 markers remained, out of which 54 markers with high map intervals and recombination frequencies were also eliminated. The 54 removed markers had as high as 63.34 cM map intervals and/or 0.6712 recombination frequencies. A total of 2648 SNPs were used to construct the linkage maps of 20 chromosomes (Supplementary Table S1) and QTL analysis. The total linkage maps spanned 3608.4 cM with a mean of 1.36 cM between markers. Chromosomes 13 (262.44 cM) and 15 (145.71 cM) had the largest and shortest linkage maps, respectively. 

**Figure 2:**
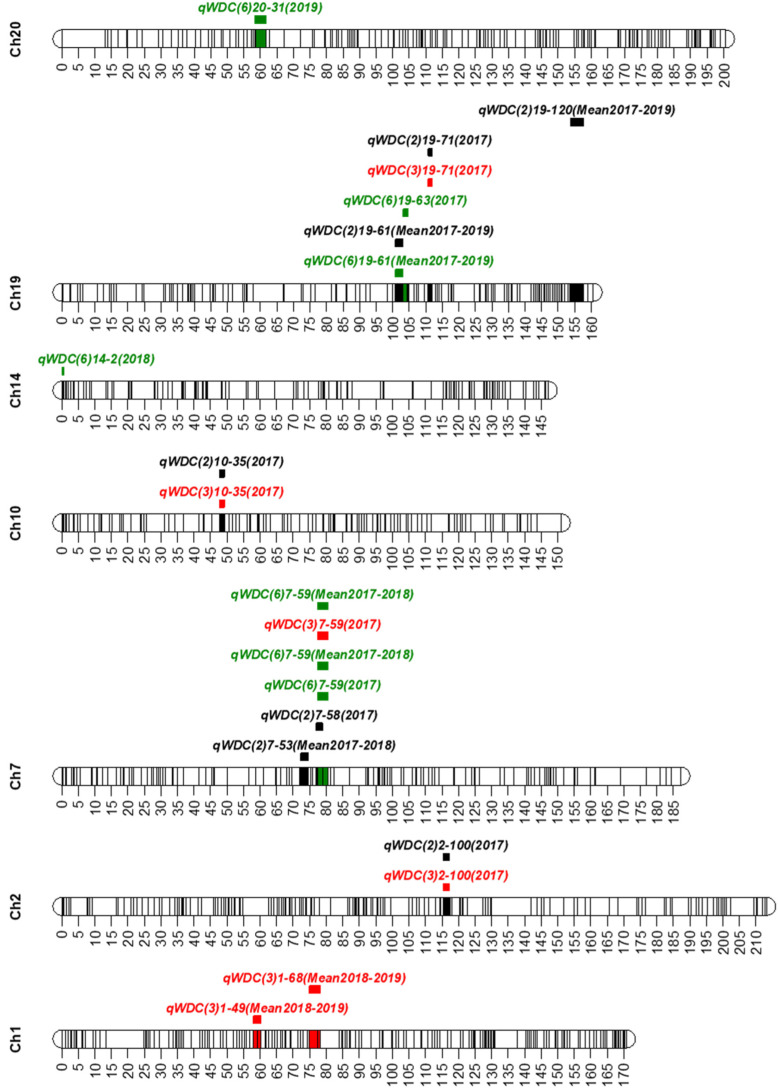
Positions of the QTLs for drought tolerance on seven chromosomes (Ch). q: QTL, WDC: weighted drought coefficient. In the QTL names, the first number in the parentheses after WDC represents the number of traits considered to calculate WDC (black, red, and green for 2, 3, and 6 traits, respectively; the second number is for chromosome name; the number after a dash (-) represents the sequential number of the marker on the linkage map; and mean denotes the average value of the traits in different years (2017–2019). The lines inside the chromosomes represent the position of markers used to construct the linkage map. The colored bars indicate the QTL regions. The scaled numbers next to chromosomes indicate the genetic length (cM) of the chromosome.

A total of 10 QTLs with a range of 3.52 to 4.71 LOD and 8.1 to 12.9% PVE were identified on seven chromosomes (1, 2, 7, 10, 14, 19, and 20). One QTL was found on five chromosomes 2, 7, 10, 14, and 20; two QTLs on chromosome 1; and three QTLs on chromosome 19 (Figure 2 and Table 2). Five QTLs—*qWDC2-1*, *qWDC7-1*, *qWDC10-1*, *qWDC19-1*, and *qWDC19-2* were detected on the different combinations of traits. These QTLs were considered to be stable QTLs for drought tolerance. Interestingly, *qWDC7-1* was detected on all three combinations of traits. *qWDC2-1* (LOD = 4.68, PVE = 10.6%), *qWDC7-1* (LOD = 4.44, PVE = 10.3%), and *qWDC19-2* (LOD = 4.57, PVE = 10.3%) which were identified on more than two trait combinations and had more than 10% PVE were considered to be stable and major QTL accounting for drought tolerance.

### 2.4. Candidate Gene Prediction

The potential candidate genes that resided within 200 kb of the QTLs were searched in Soybase (www.soybase.org, accessed on 20 April 2021), NCBI (https://www.ncbi.nlm.nih.gov/, accessed on 20 April 2021), and Phytozyme (https://phytozome.jgi.doe.gov, accessed on 20 April 2021).Twelve potential candidate genes were found within the 200 kb of the QTL regions (Table 3). Four genes—*Glyma07g10321*, *Glyma07g10340*, *Glyma07g10440*, and *Glyma07g11470*—reside in one of the major stable QTL *qWDC7-1*. They are related to myeloblastosis (MYB) transcription factor family, a leucine-rich repeat receptor-like protein kinase, calmodulin binding protein-like, and mitogen-activated protein kinase, respectively. Gene *Glyma01g04710* is related to glutathione S-transferase (GST). A few genes, such as *Glyma19g33750*, *Glyma19g34210*, and *Glyma20g22311* are found to be directly associated with a stress response.

## 3. Discussion

The drought tolerance mechanism in plants is highly complex and is an outcome of complicated networks of multiple genes. Various physiological and biochemical alterations, due to drought stress, have been identified in soybean plants [9,10,11,12] that may visibly reflect in traits like PH, NN, BN, PN, BM, and LA [13,14,15]. Qi et al. [29] found a significant correlation between comprehensive drought resistance coefficient and WDC which was calculated by considering 35 morphological, physiological, and biochemical indicators including plant height and aboveground dry weight (biomass), which were also considered in the present study. These two traits (plant height and aboveground dry weight (biomass) incorporated in the previous report [29] were significantly correlated with other traits considered in the present study. As most of these six traits were significantly correlated (Supplementary Table S2), an integrated parameter WDC, derived from these traits, could appropriately represent them whilst analyzing the QTL for drought tolerance. Similarly, positive correlations of the number of nodes and pods with seed yield [30] as well as the associations of leaf area distribution with biomass and thereby with the number of pods, seed number, and seed yield [31] have been reported in soybean under low water availability, indicating the potential application of the QTL results of the present study in the soybean seed yield under drought condition.

The consistently higher WDC (Table 1) value of ‘PI416937’ than that of ‘Cheonsang’ over three years showed the former parent is better drought-tolerant than the latter one. Wide range and continuous variations in WDC value of RILs across different environments (year) indicated a quantitative nature of WDC, suggesting the appropriateness of choosing these parents to develop the RIL population for QTL analysis. The transgressive segregation of the genotypes having WDC beyond either parent could be exploited in breeding for drought tolerance [32]. Although high broad-sense heritabilities for six traits were observed in individual years (up to 0.90), the mean year data showed relatively low heritability (up to 0.42) (Supplementary Table S3), suggesting a substantial influence of growing environment on the traits. The highly significant (*p* < 0.0001) genotype × year interaction also indicated the major influence of environment on the traits (Supplementary Table S4). 

The chromosomal locations of seven QTLs identified in this study overlapped or positioned adjacent to related QTLs and/or potential candidate genes reported earlier, whereas two QTLs (*qWDC1-1* and *qWDC1-2*) on chromosome 1 and one QTL (*qWDC19-3*) on chromosome 19 were new. *qWDC2-1* was located nearby Satt266 (< 260 kb) that linked to a QTL for canopy wilting [19]. Another QTL *MPW2.2* (Gm02_14594196) for drought tolerance [25] was also located near to (< 33 kb) *qWDC2-1*. *qWDC7-1* was overlapped its position with the QTLs *qPH28-M-1* and *qPH-B2-1* for plant height [33,34] and the QTL *qPN-M-1* for pod number [35]. *qWDC10-1* was colocalized the physical position with the QTLs *MPW10.5* (Gm10_38212261) for drought tolerance [25], *qPH49-O-1* for plant height [33], and *qPN-O-1* for pod number [36]. A QTL *qPH-B2-1* for plant height [34] located within 300 kb from *qWDC14-1* identified on chromosome 14. *qWDC19-1* and *qWDC19-2* were colocalized with the QTLs *qPH07-L-1* and *qPH-L-2*, respectively, for plant height [33,34] and *qPN-L-1* for pod number [36], and located within the QTL *MPH19.2* for drought tolerance [25]. Similarly, the QTL *qWDC20-1* on chromosome 20 was overlapped with a QTL *qPN-I-1* for pod number [36]. 

Several biochemical mechanisms and genes might be involved in stress tolerance in soybean [37]. *Glyma01g04710* related to GST was found to be resided in the QTL *qWDC1-1*. GSTs play multiple roles in plants including drought stress response in *Arabidopsis* [38], rice [39], and soybean [40]. Over-expression of a GST gene, *GsGST*, from wild soybean (*Glycine soja*) enhances drought and salt tolerance in transgenic tobacco [41]. Overexpression of soybean BiP (binding protein), a molecular chaperon, similar to *Glyma01g04750* in QTL *qWDC1-1*, can enhance drought tolerance in soybean [42]. 

The products of four genes—*Glyma07g10321*, *Glyma07g10340*, *Glyma07g10440*, and *Glyma07g11470*—in the QTL region of chromosome 7 are related to the regulation of drought stress in soybean and other plants. For instance, *Arabidopsis* calmodulin-binding transcription factor CAMTA1 is involved in drought stress response [43]. GmMYB84, a novel MYB confers drought tolerance in soybean [44]. Overexpression of the leucine-rich receptor-like kinase gene *LRK2* increases drought tolerance and tiller number in rice [45]. Expression of a truncated ERECTA (a gene family encoding leucine-rich repeat receptor-like kinase) protein modified the growth and abiotic stress tolerance in soybean [46]. Morever, mitogen-activated protein kinase positively regulates drought stress in tomato [47]. 

In the QTL region of chromosome 19, four candidate genes were found. *Glyma19g33750* is associated with salt stress response and *Glyma19g34210* is related to a heat shock transcription factor. The other two genes—*Glyma19g33650* and *Glyma19g34550*—are linked with glutathione peroxidase and Golgi SNARE Bet1-related, respectively. Heat stress transcription factors play a crucial role in plants’ response to several abiotic stresses by regulating the expression of stress-responsive genes, such as heat shock proteins [48]. Overexpression of a glutathione peroxidase 5 (*RcGPX5*) gene increases drought tolerance in *Salvia miltiorrhiza* [49]. Furthermore, reactive oxygen species scavenging activities, including glutathione peroxidase, increased in soybean plants and were positively correlated with seed yield under drought stress [50]. Similarly, SNAREs are found to play a role in plant drought tolerance [51]. 

The QTLs for drought tolerance, which were identified considering up to six traits, were either colocalized or positioned adjacent to the previously reported QTLs and/or potential candidate genes associated with stresses and/or the traits of consideration. It increased the reliability of the QTL and the results could provide a valuable reference for the molecular marker-assisted selection and further fine-mapping of genes for drought tolerance.

## 4. Materials and Methods

### 4.1. Plant Material and Growing Conditions

A RIL population developed through the single seed descent method from a cross between a drought-tolerant ‘PI416937’ and susceptible ‘Cheonsang’ cultivar was used to analyze the QTL for drought tolerance. The parents and 140 RILs of F_6:7_, F_6:8_, and F_6:9_ were grown in plastic houses at the Department of Southern Area Crop Science, Daegu (35°54′24″ N 128°26′51″ E) in 2017 and Miryang (35°29′32″ N 128°44′35″ E), Korea in 2018 and 2019. The plastic house was a kind of rain shelter with the ambient environmental condition. Soybean seedlings were grown in the seedling-growing plastic trays and then healthy uniform seedlings at the first trifoliate stage (V1) were transplanted in the plastic houses. Three to five plants of each genotype were transplanted in the plastic house at 30 cm row to row and plant to plant distance in two replications for control and drought stress each. Irrigation was applied through drip irrigation and drought stress was imposed from the V4 to R4 stages by withholding irrigation during the period. The plants in the control plots were regularly irrigated to avoid drought stress. 

### 4.2. Measurement of Soil Moisture Content

The soil moisture content of the control and drought-stressed plots was measured using a soil moisture meter (TDR 300, Spectrum Technologies, Plainfield, IL, USA).

### 4.3. Measurement of Traits and Phenotyping

The plant height, number of nodes and branches on the main stem, number of pods, and leaf area were measured at the R6 stage, whereas the biomass (including seeds) was measured when plant was harvested at the R8 stage. The traits were measured in three to five plants of each replication. Leaf area was measured using the Easy Leaf Area software [52]. 

Each of drought coefficient (DC) value of six traits was calculated as the ratio of individual trait under the drought to control conditions as shown in the equation below.

$$DC=Trait_{Drought}/Trait_{Control}$$

The weighted drought coefficient (WDC) was calculated as follows [29]. This is one of the methods of comprehensive evaluation of drought tolerance in soybean that were identified from eight yield-related agronomic traits, and rigorous studies of different evaluation methods by establishing a relative correlation with the traits.

$$WDC=\overset{n}{\underset{i=1}{\sum }}[DC\times (\left|ri\right|÷\overset{n}{\underset{i=1}{\sum }}\left|ri\right|)]$$

where *DC* is mean drought coefficient of the traits considered, *r* is the correlation coefficient of the mean *DC* of the traits considered and the *DC* of individual traits. 

The QTLs for drought tolerance were analyzed by considering the WDC values calculated from the combination of two (biomass and leaf area), three (plant height, biomass, and leaf area), and six (plant height, number of nodes, number of branches, number of pods, biomass, and leaf area) traits.

### 4.4. DNA Extraction and Genotyping

Genomic DNA was extracted from the young trifoliate leaves using a kit (Exgene^TM^ Plant SV Miniprep Kit, GeneAll, Seoul, Korea) as described in a previous report [53]. The parents and RILs were genotyped using a 180K Axiom^®^ SoyaSNP array [54].

### 4.5. Construction of Linkage Map and QTL Analysis

The polymorphic markers between the parents were separated from the 180K SNPs and subjected to screen for redundancy. In the genetic study, the redundant markers can make no additional information because they have identical segregation in the genetic population and show clustering at one genetic position in the linkage map construction [55]. Therefore, the redundant markers were separated out using the Bin function before the linkage map construction using the Map function in IciMapping V4.1 [56]. The algorithms set for the Bin function were as follows: significant distortion of *p* < 0.001 and missing data with >15%. The linkage map was constructed using the Kosambi mapping function following the manufacturer’s instruction with the adjusted parameters: grouping by 3.0 logarithm of odds (LOD) threshold, ordering by nnTwoOpt, and rippling by the sum of adjacent recombination fractions. The SNPs with high map intervals and recombination frequencies were further removed.

QTLs were analyzed with the composite interval mapping (CIM) using QTL Cartographer V2.5 (available at https://brcwebportal.cos.ncsu.edu/qtlcart/WQTLCart.htm, 5 March 2021) following the manufacturer’s instructions with adjusted parameters: Model 6, forward and backward regression, walk speed of 1.0 cM, and putative QTL with a window size of 10 cM. The number of control markers was 5, which was a default parameter. The LOD threshold for each trait was determined using a 1000 permutation test at *p* < 0.05. After the completion of the analysis, the QTL information was extracted by adjusting a minimum of 10 cM between QTL and 2-LOD support intervals. The graphical presentation of linkage maps with QTLs was done using MapChart 2.32 [57].

The QTLs were named by combing abbreviated letters *q* for QTL and *WDC* for weighted drought coefficient followed by the name of chromosome and nth QTL on the chromosome. For instance, *qWDC1-2* denotes the second QTL identified on chromosome 1.

### 4.6. Potential Candidate Genes Prediction

Potential candidate genes were searched within 200 kb regions of QTLs. The genes, which were directly linked to drought stress response and/or associated with the stress, were considered candidate genes. The name and function of drought stress-related potential candidate genes that resided in the QTLs were searched in Soybase (www.soybase.org), NCBI (https://www.ncbi.nlm.nih.gov/), and Phytozyme (https://phytozome.jgi.doe.gov). The Glyma1.1 gene version was used to collect the gene information.

### 4.7. Data Analysis

Analysis of variance (ANOVA) and Pearson’s correlation were calculated in SAS9.4 using PROC GLM and PROC CORR, respectively. Broad-sense heritability (h^2^) was determined as the ratio of genotypic variance (*σ*^2^*_G_*) to phenotypic variance (*σ*^2^*_P_*) as described earlier [58]. The genotypic variance (*σ*^2^*_G_*) component was estimated as: *M*_3_−M_2_/*rY* where *M*_3_ is the mean square of genotype, *M*_2_ is the mean square of genotype × year, *r* is the number of replications, and *Y* is the number of years. The phenotypic variance (*σ*^2^*_P_*) component was estimated using the equation *σ*^2^*_P_* = $\sigma_{G}^{2}+\sigma_{GY}^{2}/Y+\sigma_{e/rY}^{2}$ where *σ*^2^*_GY_* and *σ*^2^*_e_* are the components of genotype × year and error variances, respectively. The component of genotype × year variance (*σ*^2^*_GY_*) was estimated as: *M*_2_−*M*_1_/*r* where *M*_1_ is the mean square of error (*σ*^2^*_e_*).

## Figures and Tables

**Table 1:** Weighted drought coefficient (WDC) of the parents and recombinant inbred lines (RILs) for three years (2017–2019) and their mean.

Trait	Year	Parents		RILs	
		PI416937	Cheonsang	Mean	Range
WDC (2)	2017	0.71	0.28	0.73	0.12–2.16
	2018	0.65	0.53	0.57	0.23–1.01
	2019	0.90	0.45	0.56	0.23–1.04
	Mean ^1^	0.76	0.42	0.62	0.12–1.04
WDC (3)	2017	0.78	0.43	0.77	0.32–1.87
	2018	0.76	0.62	0.65	0.36–0.99
	2019	0.86	0.51	0.62	0.31–1.02
	Mean	0.80	0.52	0.68	0.31–1.87
WDC (6)	2017	0.76	0.45	0.86	0.22–3.06
	2018	0.78	0.63	0.67	0.38–1.01
	2019	0.82	0.62	0.70	0.33–1.15
	Mean	0.79	0.57	0.74	0.22–3.06

^1^ Average value of three years. The values in the parentheses after WDC indicate the number of traits considered to calculate WDC: 2 (biomass and leaf area), 3 (plant height, biomass, and leaf area), and 6 (plant height, node number, branch number, pod number, biomass, and leaf area).

**Table 2:** QTLs for drought tolerance identified in a recombinant inbred line population derived from a drought-tolerant ‘PI416937’ and susceptible ‘Cheonsang’ parents.

QTL Name ^1^	Traits ^2^	Chr(LG) ^3^	Genetic Position (cM)	Year	Marker Interval	Physical Position of Markers ^4^ (bp)	LOD ^5^	PVE ^6^ (%)	Add ^7^
*qWDC1-1*	3	1(D1a)	59.0	Mean (2018–2019)	AX-90430153–AX-90472468	4,523,676–5,311,697	3.59	9.0	0.0299
*qWDC1-2*	3	1(D1a)	77.5	Mean (2018–2019)	AX-90491463–AX-90348846	8,574,531–12,537,020	3.88	9.5	0.0303
*qWDC2-1*	3	2(D1b)	116.3	2017	AX-90446012–AX-90363541	14,349,986–14,561,578	4.53	10.3	0.0998
*qWDC2-1*	2	2(D1b)	116.3	2017	AX-90446012–AX-90363541	14,349,986–14,561,578	4.68	10.6	0.1276
*qWDC7-1*	2	7(M)	73.1	Mean (2017–2018)	AX-90361948–AX-90313028	8,428,091–8,490,557	3.51	8.2	−0.0539
*qWDC7-1*	2	7(M)	78.3	2017	AX-90524222–AX-90514687	8,678,861–9,630,217	3.90	8.7	−0.1102
*qWDC7-1*	6	7(M)	78.9	2017	AX-90395090–AX-90450726	9,458,480–10,232,736	3.58	8.5	−0.1403
*qWDC7-1*	6	7(M)	78.9	Mean (2017–2018)	AX-90395090–AX-90450726	9,458,480–10,232,736	3.57	8.7	−0.0711
*qWDC7-1*	3	7(M)	78.9	2017	AX-90395090–AX-90450726	9,458,480–10,232,736	4.44	10.0	−0.0935
*qWDC7-1*	3	7(M)	78.9	Mean (2017–2018)	AX-90395090–AX-90450726	9,458,480–10,232,736	4.42	10.3	−0.0489
*qWDC10-1*	3	10(O)	48.5	2017	AX-90408464–AX-90377420	38,465,737–38,164,877	4.14	9.2	−0.0910
*qWDC10-1*	2	10(O)	48.5	2017	AX-90408464–AX-90377420	38,465,737–38,164,877	4.47	9.9	−0.1189
*qWDC14-1*	6	14(B2)	0.4	2018	AX-90403945–AX-90517018	241,228–369,721	3.65	9.6	−0.0405
*qWDC19-1*	6	19(L)	101.5	Mean (2017–2019)	AX-90425812–AX-90334270	41,077,065–41,940,539	3.54	8.4	0.0467
*qWDC19-1*	2	19(L)	101.5	Mean (2017–2019)	AX-90425812–AX-90334270	41,077,065–41,940,539	3.52	8.1	0.0397
*qWDC19-1*	6	19(L)	104.2	2017	AX-90334270–AX-90311493	41,940,539–42,045,317	4.03	9.6	0.1491
*qWDC19-2*	3	19(L)	111.5	2017	AX-90480787–AX-90489545	43,473,467–43,030,013	4.45	10.0	0.0938
*qWDC19-2*	2	19(L)	111.5	2017	AX-90480787–AX-90489545	43,473,467–43,030,013	4.57	10.3	0.1203
*qWDC19-3*	2	19(L)	156.9	Mean (2017–2019)	AX-90403789–AX-90364479	49,436,986–49,727,405	3.91	9.1	−0.0425
*qWDC20-1*	6	20(I)	58.7	2019	AX-90318489–AX-90405719	30,153,192–32,595,196	4.71	12.9	0.0601

^1^ QTL detected at the same, adjacent or overlapping marker intervals were considered the same QTL. ^2^ Number of traits (2: biomass and leaf area, 3: plant height, biomass, and leaf area, and 6: plant height, node number, branch number, pod number, biomass, and leaf area) considered to analyzed the QTL. ^3^ Chromosome (Chr) and linkage group (LG). ^4^ Physical position of the marker interval. The soybean reference genome (*Glycine max* Wm82.a1) was used to determine the physical position of the markers. ^5^ Logarithm of odds value at the peak likelihood of QTL. ^6^ Phenotypic variation explained by the QTL. ^7^ Additive effect, a positive value indicates that ‘PI416937’ contributed the allele, and negative value indicates that ‘Cheonsang’ contributed the allele for the PVE.

**Table 3:** Potential candidate genes related to stress tolerance that resided within 200 kb of the QTL regions.

SN	Gene Name	Physical Location (bp)	QTL	Gene Description
1	*Glyma01g04710*	4,323,774–4,325,439	*qWDC1-1*	Glutathione S-transferase, GST, Superfamily, GST domain containing
2	*Glyma01g04750*	4,367,349–4,370,072	*qWDC1-1*	Molecular chaperone (DnaJ superfamily)
3	*Glyma01g09515*	11,687,515–11,688,465	*qWDC1-2*	Growth factor activity
4	*Glyma07g10321*	8,632,573–8,633,965	*qWDC7-1*	MYB-like DNA-binding protein MYB
5	*Glyma07g10340*	8,640,435–8,642,843	*qWDC7-1*	Leucine-rich repeat receptor-like protein kinase
6	*Glyma07g10440*	8,726,024–8,730,618	*qWDC7-1*	Calmodulin-binding protein-like
7	*Glyma07g11470*	9,651,174–9,657,518	*qWDC7-1*	Mitogen-activated protein kinase
8	*Glyma19g33650*	41,237,434–41,241,804	*qWDC19-1*	Glutathione peroxidase
9	*Glyma19g33750*	41,345,217–41,346,003	*qWDC19-1*	Salt stress response/antifungal
10	*Glyma19g34210*	41,826,839–41,830,086	*qWDC19-1*	Heat shock transcription factor
11	*Glyma19g34550*	42,144,502–42,144,657	*qWDC19-1*	Golgi SNARE Bet1-related
12	*Glyma20g22311*	32,330,953–32,332,259	*qWDC20-1*	Stress responsive protein

The name and description of the drought stress-related potential candidate genes were searched in Soybase (www.soybase.org), NCBI (https://www.ncbi.nlm.nih.gov/), and Phytozyme (https://phytozome.jgi.doe.gov).

## Supplementary Materials

The following are available online at https://www.mdpi.com/article/10.3390/plants10091816/s1, Table S1: Marker distribution and length of linkage maps of 20 chromosomes, Table S2: Correlation between different traits under control and drought conditions, Table S3: Plant height (PH), number of nodes on main stem (NN), number of branches on main stem (BN), number of pods (PN), biomass (BM), and leaf area (LA) under the control (C) and drought (D) in three years, Table S4: Analysis of variance for plant height, number of nodes and branched in main stem, number of pods, biomass, and leaf area of the recombinant inbred line (RIL) population derived from a drought-tolerant ‘PI416937’ and susceptible ‘Cheonsang’ parents.
