# Supplementary material for: Quantitative Trait Locus Mapping for Drought Tolerance in Soybean Recombinant Inbred Line Population
Source: Plants (Basel). 2021 Aug 31;10(9):1816. doi: 10.3390/plants10091816 (PMC8471639; doi:10.3390/plants10091816)
Supplement: Supplementary file 1 [file plants-10-01816-s001.zip › plants-1307436-supplementary.pdf]

Quantitative trait locus mapping for drought tolerance in soybean recombinant inbred line population

Sanjeev Kumar Dhungana [1], Ji-Hee Park [1,*], Jae-Hyeon Oh [2], Beom-Kyu Kang [1], Jeong-Hyun Seo [1], Jung-Sook Sung [1], Hong-Sik Kim [3], Sang-Ouk Shin [1], In-Yeol Baek [1] and Chan-Sik Jung [1]

1 Upland Crop Breeding Research Division, Department of Southern Area Crop Science, National Institute of Crop Science, Rural Development Administration, Miryang 50424, Republic of Korea; sanjeev@korea.kr (S.K.D), heeya91@korea.kr (J.-H.P.), hellobk01@korea.kr (B.-K.K.), next0501@korea.kr (J.-H.S.), sjs31@korea.kr (J.-S.S.), shinso32@korea.kr (S.-O.S.), baekiy@korea.kr (I.-Y.B.), jung100@korea.kr (C.-S.J.)

2 Gene Engineering Division, Department of Agricultural Biotechnology, National Institute of Agricultural Sciences, Rural Development Administration, Jeonju 54874, Republic of Korea; jhoh8288@korea.kr

3 Crop Post-Harvest Technology Division, Department of Central Area Crop Science, National Institute of Crop Science, Rural Development Administration, Suwon 16429, Republic of Korea; kimhongs@korea.kr

* Correspondence: heeya91@korea.kr; Tel.: (+82-55-350-1234)

Name of Journal: Plants

Supplementary Table S1. Marker distribution and length of linkage maps of 20 chromosomes

Name of chromosome	Number of markers	Linkage map length (cM)	Average distance between adjacent markers (cM)
Chromosome1	158	170.8	1.08
Chromosome2	153	213.18	1.39
Chromosome3	177	213.65	1.21
Chromosome4	97	170.24	1.76
Chromosome5	87	153.82	1.77
Chromosome6	122	199.05	1.63
Chromosome7	122	187.31	1.54
Chromosome8	113	211.63	1.87
Chromosome9	184	192.17	1.04
Chromosome10	110	151.08	1.37
Chromosome11	87	146.01	1.68
Chromosome12	89	157.56	1.77
Chromosome13	209	262.44	1.26
Chromosome14	108	147.19	1.36
Chromosome15	64	145.71	2.28
Chromosome16	214	190.82	0.89
Chromosome17	85	145.84	1.72
Chromosome18	196	188.72	0.96
Chromosome19	123	160.61	1.31
Chromosome20	150	200.57	1.34
Whole Genome	2648	3608.4	1.36

Supplementary Table S2. Correlation between different traits under control and drought conditions

Trait	Treatment	Plant height	Node number	Branch number	Pod number	Biomass	Leaf area
Plant height	Control	1					
	Drought	1					
Node number	Control	0.595^{****}	1				
	Drought	0.744^{****}	1				
Branch number	Control	0.174^{*}	0.155^{ns}	1			
	Drought	0.337^{****}	0.294^{***}	1			
Pod number	Control	0.192^{*}	0.445^{****}	0.361^{****}	1		
	Drought	0.287^{***}	0.281^{***}	0.386^{****}	1		
Biomass	Control	0.221^{**}	0.452^{****}	0.209^{*}	0.851^{****}	1	
	Drought	0.389^{****}	0.379^{****}	0.209^{*}	0.811^{****}	1	
Leaf area	Control	0.100^{ns}	0.022^{ns}	-0.135^{ns}	0.186^{*}	0.360^{****}	1
	Drought	0.107^{ns}	-0.027^{ns}	0.106^{ns}	0.105^{ns}	0.281^{***}	1

^{ns}, *, **, ***, and **** denote non-significant and significant difference at <.05, <.01, <.001, and <.0001, respectively.

Supplementary Table S3. Plant height (PH), number of nodes on main stem (NN), number of branches on main stem (BN), number of pods (PN), biomass (BM), and leaf area (LA) under the control (C) and drought (D) in three years

Trait	Year	Parents				RILs				H^{2} [2]	
		PI416937		Cheonsang		Mean		Range			
		C	D	C	D	C	D	C	D	C	D
PH	2017	82.80	83.00	81.45	81.00	94.31	85.85	40.20–144.50	50.00–147.5	0.58	na
	2018	37.00	35.67	43.00	35.67	37.44	28.59	25.00–51.33	17.00–41.67	0.89	0.85
	2019	48.67	37.00	65.00	42.00	60.02	45.87	35.33–99.67	18.00–80.33	0.89	0.86
	Mean [1]	56.16	51.89	63.15	52.89	64.12	53.06	25.00–144.50	17.00–147.50	0.33	0.40
NN	2017	15.00	16.00	14.00	15.00	14.72	15.11	8.00–20.00	9.00–19.00	0.66	na[b]
	2018	9.67	10.00	11.00	10.00	10.38	9.27	8.00–12.67	7.00–12.00	0.64	0.64
	2019	13.00	11.00	13.00	11.50	12.88	12.52	9.00–16.33	6.00–18.00	0.75	0.79
	Mean	12.56	12.33	12.67	12.17	12.68	12.27	8.00–20.00	6.00–19.00	0.42	0.20
BN	2017	na [3]	na	na	na	na	na	na	na	na	na
	2018	3.67	1.67	3.33	1.33	2.71	1.17	0.67–5.00	0.00–3.67	0.71	0.77
	2019	3.67	2.67	2.00	1.00	3.75	2.76	0.00–6.67	0.00–6.50	0.59	0.68
	Mean	3.67	2.17	2.67	1.17	3.23	1.97	0.00–6.67	0.00–6.50	0.36	0.30
PN	2017	161.5	118.00	81.5	36.00	67.79	49.17	0.00–206.00	2.00–168.00	0.53	na
	2018	17.67	19.67	27.00	19.00	24.22	12.04	9.33–51.33	2.00–31.33	0.83	0.79
	2019	52.33	42.33	60.00	48.50	53.90	33.57	7.00–140.67	1.33–120.00	0.74	0.73
	Mean	77.17	60.00	56.17	34.50	48.81	31.44	0.00–206.00	1.33–168.00	0.27	0.01
LA	2017	119.63	105.00	121.76	36.47	96.53	58.05	19.11–165.97	11.81–132.2	0.53	0.34
	2018	62.72	39.43	59.11	28.57	53.86	27.92	28.40–83.38	8.53–72.10	0.82	0.84
	2019	37.68	34.79	72.26	28.81	51.61	25.75	14.87–134.74	7.91–80.69	0.89	0.90
	Mean	73.34	60.07	84.38	31.28	67.43	37.14	14.87–165.97	7.91–106.10	0.36	0.16
BM	2017	480.50	303.00	247.50	68.00	189.2	127.11	18.00–542.00	12.00–498.00	0.49	0.41
	2018	33.33	20.17	37.43	19.83	31.87	14.17	12.40–65.10	2.70–36.77	0.78	0.83
	2019	21.05	48.83	21.64	50.50	80.61	43.24	13.67–237.83	8.50–50.50	0.77	0.77
	Mean	189.73	124.00	128.09	46.11	58.65	34.37	6.00–237.83	2.70–150.50	0.26	0.17

[1] Average value of three years. [2] Broad-sense heritability. [3] Not available.

Supplementary Table S4. Analysis of variance for plant height, number of nodes and branched in main stem, number of pods, biomass, and leaf area of the recombinant inbred line (RIL) population derived from a drought-tolerant 'PI416937' and susceptible 'Cheonsang' parents

Source of Variation	Plant height		Node number		Branch number		Pod number		Biomass		Leaf area	
	F value	P value	F value	P value	F value	P value	F value	P value	F value	P value	F value	P value
RIL (G)	14.87	<.0001	9.41	<.0001	5.87	<.0001	7.66	<.0001	6.52	<.0001	10.21	<.0001
Year (Y)	7428.04	<.0001	2968.5	<.0001	203.16	<.0001	609.91	<.0001	445.64	<.0001	1515.81	<.0001
Treatment (T)	869.32	<.0001	29.76	<.0001	403.22	<.0001	414.86	<.0001	465.21	<.0001	3050.27	<.0001
G*Y	8.69	<.0001	5.66	<.0001	3.22	<.0001	5.77	<.0001	4.85	<.0001	6.38	<.0001
G*T	3.11	<.0001	2.83	<.0001	1.2	0.0634	2.33	<.0001	1.46	0.0007	3.04	<.0001
G*Y*T	3.54	<.0001	2.96	<.0001	1.61	<.0001	2.37	<.0001	1.62	<.0001	3.45	<.0001
